# Editorial: Exposome: the cutting edge knowledge in the exposure science

**DOI:** 10.3389/fpubh.2023.1225097

**Published:** 2023-11-28

**Authors:** Kelly Polido Kaneshiro Olympio

**Affiliations:** Department of Environmental Health, School of Public Health, University of São Paulo, São Paulo, Brazil

**Keywords:** exposome, exposure science, community-exposome, multi-omics analysis, outcome pathways networks

“*Exposome: the cutting edge knowledge in the exposure science*” is a Research Topic proposed to bring together manuscripts dealing with different exposures in the same epidemiological design leading to the emergence of the exposome concept. One mini review, four papers type “original research”, and one paper type “brief research report” compose this Research Topic.

Ferrante et al. summarize the current knowledge on exposome-based approaches in pediatric respiratory health area. Further, it explores practical implementation, associated evidence gaps, research limitations and future research perspectives. The authors emphasize that collaboration, sustainability, and large-scale population-based studies are needed in order to improve exposome research. Such ongoing studies also need to be continuously and systematically evaluated. In addition, huge and expensive efforts are required in terms of consortia building and of development and validation of measurement devices and statistical tools.

Anesti et al. show the utility of exposome-wide association studies (EWAS) toward understanding the relationships among the multiple factors that determine human exposure and the underlying biology, reflected as omics markers of effect on neurodevelopment during childhood. In this paper, the reader can find results from PHIME and HERACLES cohort studies. A critical methodological finding of the HERACLES study is that the simultaneous evaluation of environmental, sociodemographic and dietary parameters gives a more comprehensive picture of the most influential factors related to neurodevelopment impairment.

Lebow-Skelley et al. bring the resulting community-exposome definition demonstrating the importance of expanding the scope of exposures beyond traditional environmental influences to include the lived experience of individuals and communities. While newer exposome definitions align more closely with this community definition, traditional exposome methods do not routinely include these factors. The authors recommend to truly capture the totality of lifetime exposures and improve human health, researchers should incorporate community perspectives into exposome research.

Wu et al. discuss that the chemical part of the exposome, including drugs, may explain the increase of health effects with outcomes such as infertility, allergies, metabolic disorders, which cannot be only explained by the genetic changes. The authors highlight that computational approaches based on network science may help to understand the complexity of drug health effects, with the aim to support drug safety assessment and show that adverse outcome pathways networks (AON) analysis allowed identifying biological events that are highly connected to drugs. Furthermore, the number of events involved in a linkage pattern with drugs is a key factor that influences information loss during monopartite network projection. Such scores have the potential to quantify the uncertainty of an event involved in an AON, and could be valuable for the weight of evidence assessment of adverse outcome pathways (AOP).

Li et al. contribute with this Research Topic with a methodology and a computer-aided probabilistic model system for calculating the exposure of a person to PM_2.5_ and NO_2_ over the whole lifetime where the person is characterized by attributes such as age, gender, place of residence and work as well as socioeconomic status. The model system contains a “life course trajectory model”, which estimates the course of the education and professional development for the past lifetime of a person, whose present socioeconomic status is known. The results of these models are combined to estimate the annual average exposure for the life years of individuals and population subgroups. The exposure is then used to estimate and monetize health impacts.

Finally, Araujo et al. performed proteomics and metabolomics analyses to investigate the impact of potentially toxic elements on the informal workers' health. A Metabolome-Wide Association Study (MWAS) was performed to search for associations between blood metabolites and exposure groups. A total of 73 metabolomic compounds and 40 proteins up or down-regulated in welders were used to perform a multi-omics analysis, disclosing seven metabolic pathways potentially disturbed by the informal work. The majority of the proteins found to be statistically up or downregulated in welders also correlated with at least one blood potentially toxic element (PTE) level, providing insights into the biological responses to PTE exposures in the informal work exposure scenario, even when the exposure levels are not very elevated.

To sum up, the large cohort studies financed to study the exposome worldwide are still ongoing and many scientists do not believe is possible to measure so many variables in the same study design. Even though we do not have many results from longitudinal studies here and this is crucial to advance in Exposure Science, this Research Topic is very promising to motivate future studies linking multiple fields of research and promoting partnerships or teams with scientific capabilities that complement each other and, consequently, advance the answers that exposure science wants to reach out.

## Author contributions

The author confirms being the sole contributor of this work and has approved it for publication.

